# Phytochemical Composition and Antibacterial Activity of Medicinal Plants Against Bacterial Pathogens Isolated From Livestock Wound Infections

**DOI:** 10.1002/mbo3.70414

**Published:** 2026-09-08

**Authors:** Safia Arbab, Hanif Ullah, Mian Sayed Khan, Khalaf F. Alsharif, Fuad M. Alzahrani, Khalid J. Alzahrani, Zhongyi Zeng, Li Ka

**Affiliations:** ^1^ Medicine and Engineering Interdisciplinary Research Laboratory of Nursing & Materials/Nursing Key Laboratory of Sichuan Province, West China Hospital, Sichuan University/West China School of Nursing Sichuan University Chengdu Sichuan China; ^2^ Lanzhou Institute of Husbandry and Pharmaceutical Sciences Chinese Academy of Agricultural Sciences Lanzhou China; ^3^ Department of Zoology University of Swabi Swabi Khyber Pakhtunkhwa Pakistan; ^4^ Department of Clinical Laboratory Sciences, College of Applied Medical Sciences Taif University Saudi Arabia; ^5^ General Administration Office, Jinjiang Campus, West China Hospital of Sichuan University/West China School of Nursing Sichuan University Chengdu Sichuan China

**Keywords:** antibacterial activity, antimicrobial resistance, livestock wound infections, medicinal plants, phytochemical screening

## Abstract

Livestock wound infections are a major challenge in veterinary medicine due to the increasing prevalence of bacterial pathogens and antimicrobial resistance. This study evaluated the phytochemical composition and antibacterial activity of selected medicinal plants traditionally used for the treatment of livestock wound infections. A total of 250 livestock wound samples were processed using standard bacteriological methods, resulting in the identification of 216 bacterial isolates. The predominant bacterial pathogens included *Staphylococcus aureus* (19.2%), *Escherichia coli* (13.6%), *Staphylococcus epidermidis* (12.0%), *Streptococcus agalactiae* (9.2%), *Salmonella Typhimurium* (9.2%), *Klebsiella pneumoniae* (8.0%), *Enterococcus faecalis* (7.6%), and *Proteus mirabilis* (7.6%). Methanol, ethanol, and aqueous extracts of *Withania somnifera, Achyranthes aspera, Verbascum sinaiticum, Aloe vera*, and *Capparis zeylanica* were evaluated for antibacterial activity using the agar well diffusion method. Phytochemical screening revealed the presence of alkaloids, flavonoids, tannins, saponins, steroids, and phenolic compounds in most plant extracts. Among the tested plants, *Withania somnifera* exhibited strong antibacterial activity against *Staphylococcus aureus*, with inhibition zones ranging from 21 to 24 mm, while *Achyranthes aspera* demonstrated notable activity against *E. coli*, producing inhibition zones of 13–17 mm. Overall, Gram‐positive bacteria were more susceptible to the plant extracts than Gram‐negative bacteria. These findings suggest that the investigated medicinal plants possess significant antibacterial potential and may serve as promising alternative therapeutic agents for the management of livestock wound infections.

## Graphical abstract

The graphical abstract visually summarizes the study's key findings: it lists the major bacterial pathogens isolated from livestock wounds (such as *S. aureus* and *E. coli*) and the medicinal plants tested (*Withania somnifera*, *Achyranthes aspera*. It highlights the phytochemicals (alkaloids, flavonoids, etc.) present in the plant extracts and shows that these extracts, particularly from *W. somnifera* and *A. aspera*, demonstrated notable antibacterial activity—with Gram‐positive bacteria being more susceptible than Gram‐negative ones. Overall, the figure conveys that these traditional plants hold promise as alternative treatments for livestock wound infections.

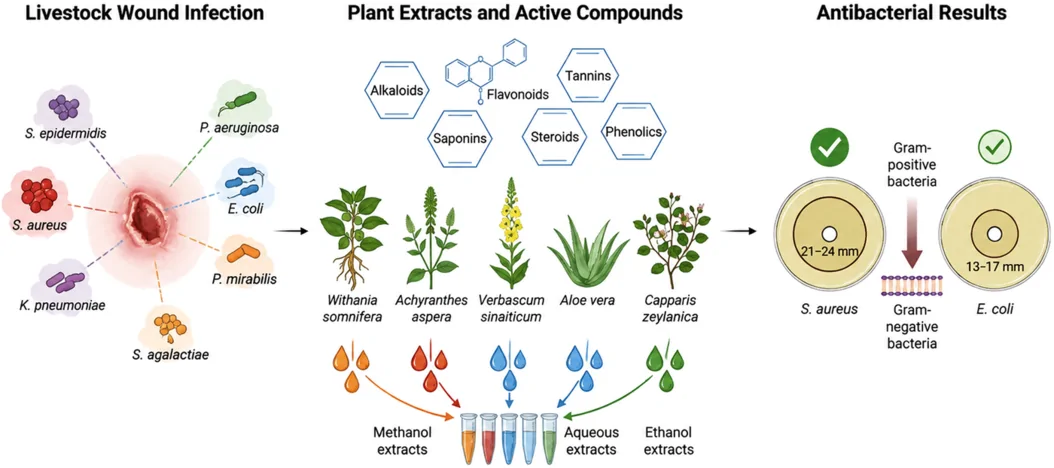

## 1 Introduction

Wound infections in livestock represent a significant challenge in animal health and production systems worldwide. These infections contribute to substantial economic losses through reduced animal performance, delayed wound healing, increased veterinary expenses, and, in severe cases, mortality. Bacterial colonization of wounds can disrupt normal tissue repair processes, leading to persistent infections, prolonged recovery periods, and decreased productivity. Common bacterial pathogens associated with livestock wound infections include *Staphylococcus aureus*, *Escherichia coli*, *Pseudomonas aeruginosa*, *Streptococcus* species, and other opportunistic microorganisms capable of causing localized and systemic infections (Arbab et al. 2025; Brooks and Brooks 2014).

Antimicrobial resistance (AMR) is a phenomenon that has been accelerated by the widespread and inappropriate use of antibiotics in human and veterinary medicine and in agricultural production. As a result, many bacterial pathogens have developed resistance to commonly used antimicrobial agents, reducing treatment effectiveness and increasing the risk of therapeutic failure. The World Health Organization (WHO) classifies AMR as one of the greatest public health challenges facing mankind, animals, food security, and environmental health (Salam 2023). As resistant bacterial strains have become more common in livestock infections, the need for alternative antimicrobial agents that are effective, affordable, and environmentally friendly has intensified (Marquardt and Li 2018; Sharma et al. 2018).

Medicinal plants have been used for centuries in traditional veterinary medicine and human medicine for treating infectious diseases and wounds. Natural products from medicinal plants are a source of a variety of secondary metabolites such as alkaloids, flavonoids, tannins, saponins, terpenoids, phenolics, and steroids, which have antimicrobial, anti‐inflammatory, antioxidant, and wound healing properties (Alamgir 2018; Hussein and El‐Anssary 2019). These phytochemicals could inhibit the growth of bacteria in several ways: disruption of bacterial cell membranes, inhibition of essential enzymes, interference with nucleic acid synthesis, and prevention of biofilm formation (Borges et al. 2016). In comparison to synthetic antibiotics, plant‐based antimicrobial agents are less harmful, more available in rural areas, and less prone to resistance (AlSheikh et al. 2020; Arbab 2022a).

Various medicinal plants traditionally used for wound treatment have been found to exhibit promising antibacterial activity against clinically important pathogens. *Withania somnifera* is known for its antimicrobial and wound healing properties, and *Achyranthes aspera* has been shown to have antibacterial properties, antioxidant, and anti‐inflammatory activity. Similarly, *Verbascum sinaiticum*, *Aloe vera*, *and Capparis zeylanica* have traditionally been considered to possess therapeutic bioactive compounds that make them useful in the treatment of skin infections and inflammatory diseases (Arbab 2021b; Srivastav et al. 2024). There have been some studies done on the phytochemical constituents and antibacterial properties of these medicinal plants against human‐associated pathogens. But little data is available on their activity against bacterial isolates recovered from the skin wound infections of animals.

The aim of this study was thus to assess the phytochemical constituents and in vitro antibacterial properties of some medicinal plants against bacterial isolates obtained from wound infections of livestock. The study also aimed to compare the antibacterial effect of the different solvent extracts and correlate the results with the phytochemical compounds found in the plants. The results of this research could inform the formulation of other plant‐based antimicrobial products for treating livestock wound infections and support additional research into the therapeutic value of these products in veterinary medicine.

## 2 Material Methods

### 2.1 Ethics Statement

This study involved the collection and analysis of the phytochemical composition and Antibacterial Activity of medicinal plants against bacterial pathogens isolated from Livestock Wound Infections. The study protocol was reviewed and approved by the Ethics Committee of the University of Swabi, Pakistan (UOS/2026/1280). All procedures were performed in accordance with relevant ethical guidelines and institutional regulations.

### 2.2 Sample Collection

A total of 250 wound swab samples were collected aseptically from livestock presenting clinical signs of infected skin wounds. Samples were collected from different livestock species (specify species) located in (specify region) during (specify collection period). Wound sites were cleaned with sterile saline solution, and wound exudates were collected using sterile cotton swabs.

The samples were immediately transferred into sterile transport tubes and transported to the laboratory under aseptic conditions. Samples were inoculated onto Mannitol Salt Agar, Blood Agar, MacConkey Agar (MAC), Eosin Methylene Blue Agar (EMB), and *Salmonella*‐*Shigella* Agar (Oxoid, UK). Plates were incubated aerobically at 37°C for 24 h.

Bacterial isolates were identified based on colony morphology, Gram staining characteristics, biochemical tests, and standard microbiological identification procedures (Arbab 2021b; Huys et al. 2002).

### 2.3 Selection and Collection of Medicinal Plants

Ethnobotanical information regarding medicinal plants traditionally used for wound healing and skin infection treatment was collected through interviews with local informants, herbal practitioners, and traditional healers following standard ethnobotanical approaches.

**Figure 1:**
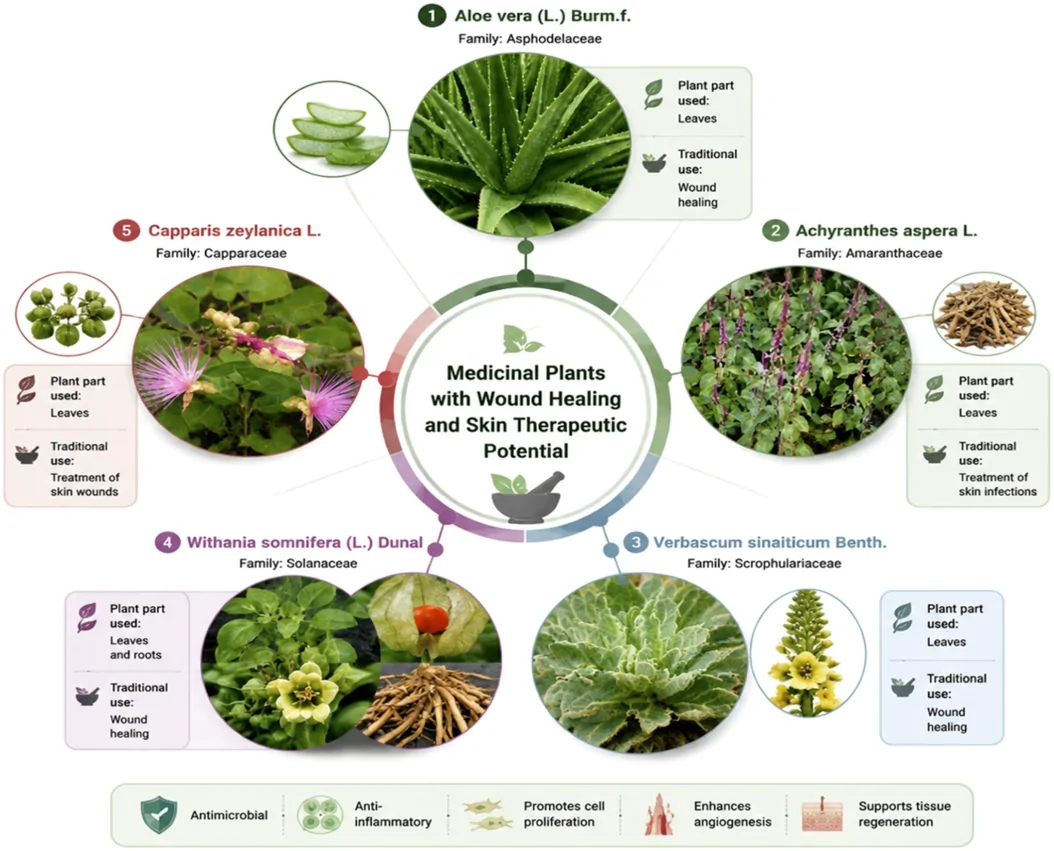
Selected medicinal plants with traditional wound‐healing and skin therapeutic applications. Schematic representation of five medicinal plants traditionally used for wound management and skin‐related disorders: (1) *Aloe vera* (L.) Burm.f. (Family: Asphodelaceae), (2) *Achyranthes aspera* L. (Family: Amaranthaceae), (3) *Verbascum sinaiticum* Benth. (Family: Scrophulariaceae), (4) *Withania somnifera* (L.) Dunal (Family: Solanaceae), and (5) *Capparis zeylanica* L. (Family: Capparaceae). The figure highlights the plant parts traditionally utilized, including leaves, roots, and other botanical components, along with their ethnomedicinal applications in wound healing and treatment of skin infections. These plants contain diverse bioactive phytochemicals with reported antimicrobial, anti‐inflammatory, antioxidant, cell‐proliferative, angiogenic, and tissue regenerative properties, supporting their potential as natural therapeutic candidates for wound repair and regenerative medicine.

Based on ethnomedicinal relevance, availability, and reported therapeutic applications, five medicinal plant species were selected for phytochemical screening and antibacterial evaluation: *Withania somnifera*, *Achyranthes aspera*, *Verbascum sinaiticum*, *Aloe vera*, and *Capparis zeylanica* (Table 1 and Figure 1).

**Table 1:** Medicinal plants selected for phytochemical screening and antibacterial evaluation.

Scientific name	Family	Plant part used	Traditional use
*Aloe vera*	Asphodelaceae	Leaves	Wound healing
*Achyranthes aspera*	Amaranthaceae	Leaves	Treatment of skin infections
*Verbascum sinaiticum*	Scrophulariaceae	Leaves	Wound healing
*Withania somnifera*	Solanaceae	Leaves and roots	Wound healing
*Capparis zeylanica*	Capparaceae	Leaves	Treatment of skin wounds

Plant materials were collected from certified nurseries and natural habitats with the assistance of local traditional practitioners. The collected plant specimens were authenticated using standard taxonomic identification keys and comparison with authenticated herbarium specimens. Voucher specimens were prepared and deposited in the Herbarium of the Microbiology Laboratory for future reference.

### 2.4 Preparation of Medicinal Plant Extract

The collected plant materials were thoroughly washed with distilled water to remove dust and contaminants and were shade‐dried at room temperature (25°C–30°C) for 10 days. The dried materials were finely powdered using an electric grinder and stored in sterile airtight containers until extraction.

Plant extraction was performed by maceration. Briefly, 50 g of powdered plant material was separately extracted using 250 mL of different solvents, including 70% ethanol, 80% methanol, and distilled water (solid‐to‐solvent ratio 1:5, w/v). The mixtures were continuously agitated on an orbital shaker for 48 h at room temperature.

Following extraction, the mixtures were filtered through Whatman No. 1 filter paper. Methanol and ethanol extracts were concentrated using a rotary evaporator (Buchi R‐300, Switzerland) at 40°C, whereas aqueous extracts were concentrated by freeze‐drying. The dried crude extracts were stored at 4°C until further analysis. For antibacterial evaluation, the extracts were reconstituted in their corresponding solvents to obtain a final concentration of 200 mg/mL.

### 2.5 Phytochemical Screening

Qualitative phytochemical screening was performed on methanol, ethanol, and aqueous extracts of the selected medicinal plants to identify major secondary metabolites, including alkaloids, flavonoids, tannins, phenolic compounds, steroids, and saponins. These phytoconstituents were evaluated due to their reported antimicrobial and therapeutic properties. The presence or absence of phytochemicals was determined using standard qualitative screening methods, as summarized in (Table 2).

**Table 2:** Summary of qualitative phytochemical screening methods and positive indicators.

Phytochemical	Test used	Positive indication
Steroids	Salkowski test	Red or brown interface
Alkaloids	Mayer's and Dragendorff's tests	Cream or reddish precipitate
Flavonoids	Alkaline reagent and Shinoda tests	Yellow or pink coloration
Saponins	Foam test	Persistent froth
Tannins/Phenolics	Ferric chloride test	Blue‐black or green coloration

#### 2.5.1 Detection of Steroids

The presence of steroids was determined using the Salkowski test. Briefly, 5 mL of plant extract was mixed with 2 mL chloroform, followed by careful addition of 2 mL concentrated sulfuric acid (H_2_SO_4_). The mixture was allowed to stand for 5 min. Formation of a reddish‐brown coloration at the interface indicated the presence of steroids (Ajiboye et al. 2013).

#### 2.5.2 Detection of Alkaloids

Alkaloids were detected using Mayer's and Dragendorff's reagents. For Mayer's test, 2 mL of plant extract was mixed with several drops of Mayer's reagent. Formation of a cream‐colored precipitate indicated the presence of alkaloids. In Dragendorff's test, addition of Dragendorff's reagent resulted in orange or reddish‐brown precipitate formation, confirming alkaloids (Ajiboye et al. 2013).

#### 2.5.3 Detection of Flavonoids

Flavonoids were detected using the alkaline reagent and Shinoda tests. For the alkaline reagent test, 2 mL of plant extract was treated with diluted sodium hydroxide solution. Development of yellow coloration, which disappeared after addition of diluted hydrochloric acid, indicated flavonoids.

For the Shinoda test, magnesium turnings and concentrated hydrochloric acid were added to the extract. Formation of pink, red, or magenta coloration confirmed the presence of flavonoids (Shaikh and Patil 2020).

#### 2.5.4 Detection of Saponins

Saponins were detected using the foam test. Plant extract was mixed with 5–10 mL distilled water and vigorously shaken for 1–2 min. Formation of persistent foam lasting several minutes indicated the presence of saponins (Godlewska et al. 2023).

#### 2.5.5 Detection of Tannins and Phenolic Compounds

Tannins and phenolic compounds were detected using ferric chloride and gelatin tests. For the ferric chloride test, 2 mL of extract was mixed with several drops of ferric chloride solution. Development of blue, green, or black coloration indicated the presence of phenolic compounds and tannins (Godlewska et al. 2023).

### 2.6 Antibacterial Activity Assay

The antibacterial activity of plant extracts was evaluated using the agar well diffusion method according to Clinical and Laboratory Standards Institute (CLSI) recommendations with slight modifications (Saleem 2015). Briefly, bacterial suspensions were standardized to 0.5 McFarland turbidity (approximately 1 × 10^8^ CFU/mL) and uniformly spread onto Mueller–Hinton agar plates using sterile cotton swabs. Wells of 6 mm diameter were prepared using a sterile cork borer.

A volume of 100 µL of each plant extract (200 mg/mL) was added into the wells. Erythromycin (15 µg/disc) was used as the positive antibiotic control, while methanol, ethanol, and distilled water were used as negative controls. The plates were allowed to stand for 30 min to facilitate extract diffusion and then incubated at 37°C for 18–24 h. Antibacterial activity was determined by measuring the diameter of inhibition zones (mm). Each experiment was performed in triplicate, and results were expressed as mean ± standard deviation.

#### 2.6.1 Preparation of Bacterial Inoculum

Pure bacterial cultures were grown on nutrient agar plates and incubated at 37°C overnight. Two to three isolated colonies were transferred into Mueller–Hinton broth and incubated until bacterial suspensions reached 0.5 McFarland turbidity. The bacterial concentration was adjusted to approximately 1 × 10^8^ CFU/mL. Turbidity was confirmed spectrophotometrically at 625 nm using a UV‐visible spectrophotometer (Arbab 2021b; Humphries et al. 2021).

#### 2.6.2 The Zone Diameter and Inhibition Activity Were Interpreted

This table describes the relationship between zone diameter (in millimeters) and inhibition activity in antimicrobial testing. The term “not seen” means there was no detectable inhibition zone. Inhibition activities are classified as: (−) = no activity; (+) = weak activity (more than 10 mm); (++) = moderate activity (10–14 mm); (+++) = strong activity (15–19 mm); (++++) = very strong activity (20–30 mm). Each phytochemical's presence or absence was denoted by (−) and (+) in (Table 3).

**Table 3:** Zone diameters for assessing inhibitory activity.

Zone diameter (mm)	Antibacterial activity
No inhibition zone	No activity (−)
< 10 mm	Low activity (+)
10–14 mm	Moderate activity (++)
15–19 mm	Strong activity (+++)
20–30 mm	Very strong activity (++++)

### 2.7 Statistical Analysis

All antibacterial experiments were performed using three independent replicates (*n* = 3), and results were expressed as mean inhibition zone diameter ± standard deviation (SD). Statistical analyses were conducted using SPSS Statistics version 25. Differences among treatment groups were analyzed using one‐way analysis of variance (ANOVA), followed by Tukey's multiple comparison test. A *p*‐value < 0.05 was considered statistically significant.

## 3 Results

### 3.1 Phytochemical Screening

**Figure 2:**
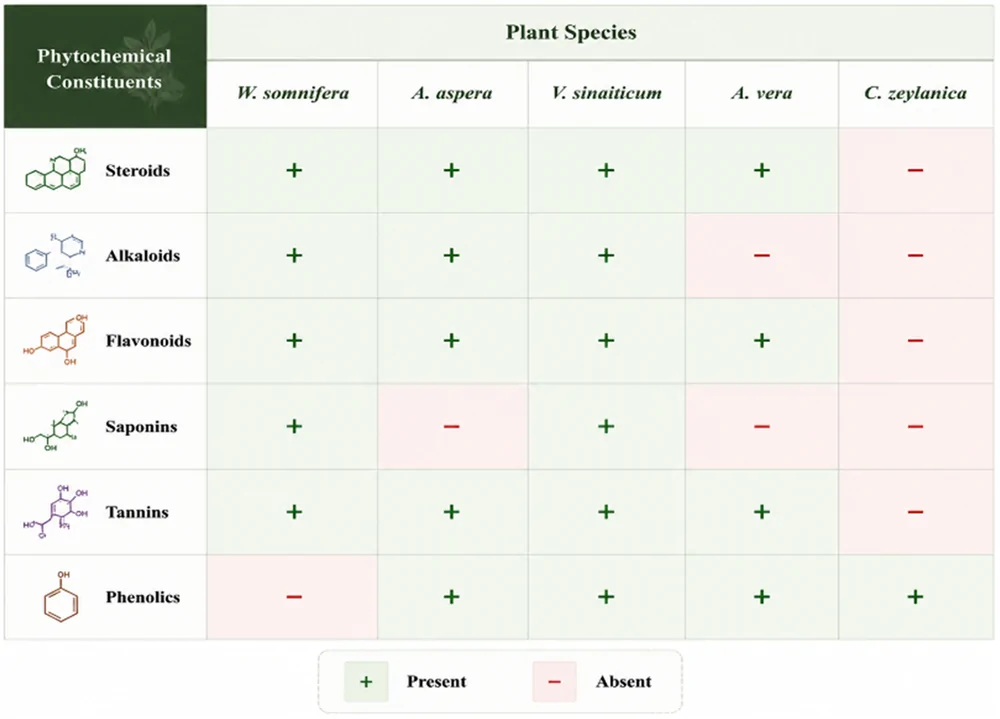
Qualitative phytochemical profiling of selected medicinal plants.

Qualitative phytochemical screening of medicinal plants' extracts revealed that there are several important bioactive compounds present in the extracts, as shown in (Figure 2). *Withania somnifera* exhibited a broad phytochemical profile, including steroids, alkaloids, flavonoids, saponins, and tannins. Other herbs, such as *Achyranthes aspera* and *Verbascum sinaiticum*, also contained several phytochemical constituents, including steroids, alkaloids, flavonoids, tannins, and phenolics.

*Aloe Vera* contains steroids, flavonoids, tannins, and phenolics, and was found to be negative for alkaloids and saponins. *Capparis zeylanica* possessed a relatively small number of phytochemicals and a positive test for only phenolic compounds. The results show the presence of bioactive secondary metabolites in these medicinal plants, which could be related to their antibacterial activity.

Presence (+) and absence (−) of major phytochemical constituents, including steroids, alkaloids, flavonoids, saponins, tannins, and phenolics, in *W. somnifera*, *A. aspera*, *V. sinaiticum*, *A. vera*, and *C. zeylanica*.

### 3.2 Bacterial Isolate Confirmation

A total of 250 wound samples were collected, with 216 samples yielding bacterial growth and 34 samples yielding no bacterial growth or mixed bacterial growth, which was not suitable for analysis.

**Figure 3:**
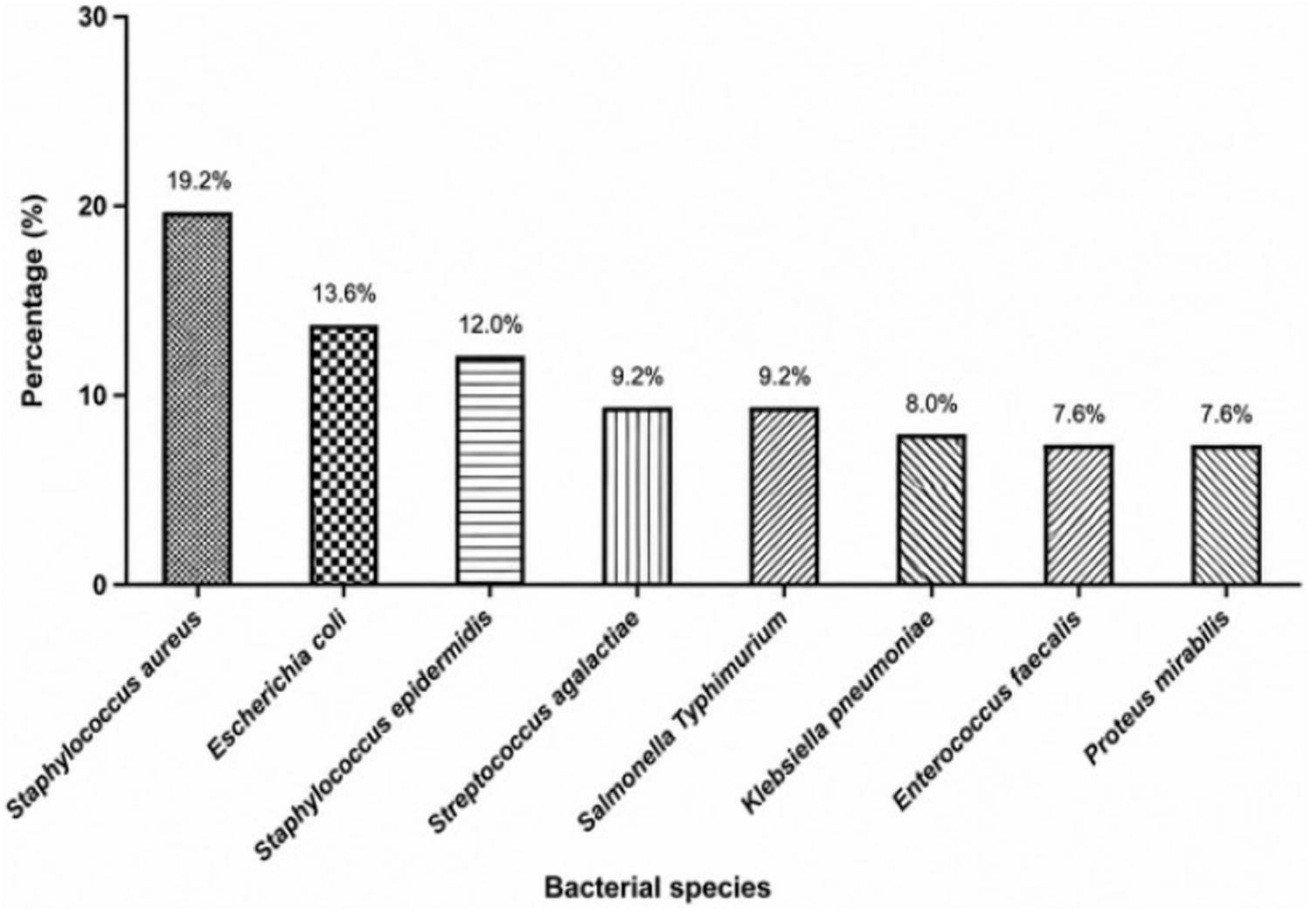
Distribution of bacterial species isolated from wound samples. The bar graph illustrates the prevalence (%) of bacterial isolates identified from wound samples, including *Staphylococcus aureus*, *Escherichia coli*, *Staphylococcus epidermidis*, *Streptococcus agalactiae*, *Salmonella Typhimurium*, *Klebsiella pneumoniae*, *Enterococcus faecalis*, and *Proteus mirabilis*. The percentage values represent the relative abundance of each bacterial species among the total isolates.

*S. aureus* was the most common bacterial isolate (19.2%), followed by *E. coli* (13.6%) and *S. epidermidis* (12.0%). The two most prevalent species were *Streptococcus agalactiae*, which represented 9.2% of isolates, and *Salmonella Typhimurium*, which represented 9.2% of the isolates. *Klebsiella pneumoniae* (8.0%) and *Enterococcus faecalis* (7.6%) accounted for the next highest numbers of isolates, followed by *Proteus mirabilis* (7.6%). As indicated in (Figure 3). In general, Gram‐positive bacteria predominated over Gram‐negative bacteria in the wound infections of livestock.

### 3.3 Antibacterial Activity of Medicinal Plant Extracts

The antibacterial potential of solvent extracts methanol, ethanol, and aqueous extracts of *Withania somnifera*, *Achyranthes aspera*, *Verbascum sinaiticum*, *Aloe vera*, and *Capparis zeylanica* against Gram‐positive and Gram‐negative bacterial strains was assessed by the agar well diffusion method.

#### 3.3.1 Antibacterial Activity Against Gram‐Positive Bacteria

**Figure 4:**
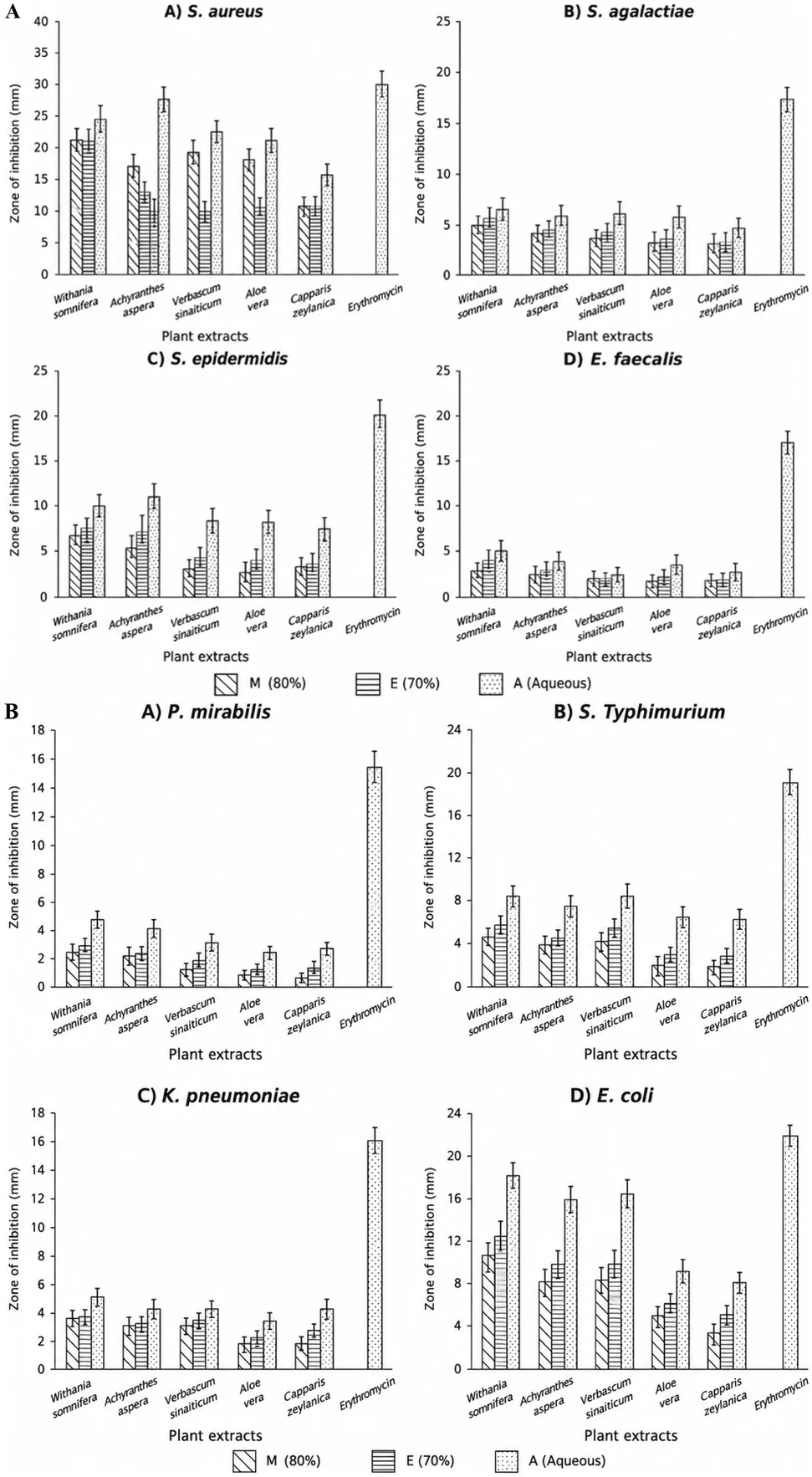
(A) Antibacterial activity of plant extracts against Gram‐positive bacterial pathogens. The figure shows the inhibitory effects of different solvent extracts (methanol, ethanol, and aqueous) from medicinal plants against A) *Staphylococcus aureus*, B) *Streptococcus agalactiae*, C) *Staphylococcus epidermidis*, and D) *Enterococcus faecalis*. The antibacterial activity was evaluated based on the zone of inhibition (mm) produced by each extract. The results demonstrate variations in antibacterial efficacy among plant species and extraction solvents, with erythromycin used as a positive control. (B) Antibacterial activity of medicinal plant extracts against Gram‐negative bacterial pathogens. The figure illustrates the inhibitory effects of methanol, ethanol, and aqueous extracts of selected medicinal plants against A) *Proteus mirabilis*, B) *Salmonella Typhimurium*, C) *Klebsiella pneumoniae*, and D) *Escherichia coli*. Antibacterial activity was evaluated by measuring the zone of inhibition (mm) produced by different plant extracts. Erythromycin was used as a positive control, demonstrating the comparative antibacterial effectiveness of the tested extracts.

The antibacterial activity of the medicinal plant extracts against Gram‐positive bacterial isolates is summarized in (Table 4A and Figure 4A). Significant variations in antibacterial activity were observed among different plant species and extraction solvents. The highest inhibitory effect against *Staphylococcus aureus* was exhibited by the aqueous extract of *Achyranthes aspera* (28.0 ± 3.6 mm), followed by the aqueous extracts of *Withania somnifera* (24.0 ± 5.5 mm) and *Verbascum sinaiticum* (23.0 ± 2.9 mm). *Withania somnifera* and *Achyranthes aspera* extracts also demonstrated comparatively higher activity against *Staphylococcus epidermidis*, whereas only weak inhibition was observed against *Streptococcus agalactiae* and *Enterococcus faecalis*. Overall, aqueous extracts showed stronger antibacterial activity than methanol and ethanol extracts against the tested Gram‐positive pathogens.

**Table 4A:** Antibacterial activity of medicinal plant extracts against Gram‐positive bacterial isolates (Mean inhibition zone ± SD, mm).

Plant Extract	Solvent	*Staphylococcus aureus*	*Streptococcus agalactiae*	*Staphylococcus epidermidis*	*Enterococcus faecalis*
*W. somnifera*	Methanol (80%)	22.0 ± 2.1	4.3 ± 1.0	7.6 ± 1.56	3.0 ± 1.15
	Ethanol (70%)	21.3 ± 6.2	4.0 ± 0.8	8.6 ± 1.56	3.3 ± 1.19
	Aqueous	24.0 ± 5.5	6.0 ± 0.9	11.0 ± 1.49	5.0 ± 1.14
*A. aspera*	Methanol (80%)	17.0 ± 4.3	4.0 ± 1.0	9.0 ± 1.63	3.0 ± 1.04
	Ethanol (70%)	12.6 ± 2.3	3.0 ± 1.0	5.0 ± 1.41	2.0 ± 0.92
	Aqueous	28.0 ± 3.6	5.0 ± 1.2	12.0 ± 1.58	5.0 ± 1.10
*V. sinaiticum*	Methanol (80%)	20.0 ± 2.4	3.0 ± 0.8	7.0 ± 1.49	2.0 ± 0.82
	Ethanol (70%)	10.0 ± 1.5	3.0 ± 0.7	3.0 ± 0.90	1.0 ± 0.50
	Aqueous	23.0 ± 2.9	5.0 ± 1.0	9.0 ± 1.33	4.0 ± 1.00
*Aloe vera*	Methanol (80%)	19.0 ± 2.2	2.0 ± 0.5	7.0 ± 1.20	2.0 ± 0.60
	Ethanol (70%)	11.0 ± 1.4	2.0 ± 0.5	2.0 ± 0.70	2.0 ± 0.50
	Aqueous	21.0 ± 2.0	5.0 ± 1.0	9.0 ± 1.10	4.0 ± 0.90
*C. zeylanica*	Methanol (80%)	11.0 ± 1.2	2.0 ± 0.4	5.0 ± 1.00	2.0 ± 0.50
	Ethanol (70%)	11.0 ± 1.0	2.0 ± 0.4	2.0 ± 0.50	2.0 ± 0.40
	Aqueous	16.0 ± 1.6	4.0 ± 0.9	8.0 ± 1.20	4.0 ± 0.80
Erythromycin	Positive control	30.0 ± 2.0	18.0 ± 1.6	20.0 ± 1.7	17.0 ± 1.5

#### 3.3.2 Activity Against Gram‐Negative Bacteria

The antibacterial effects of plant extracts against Gram‐negative bacterial isolates are presented in Table 4B and Figure 4B. Compared with Gram‐positive bacteria, Gram‐negative isolates exhibited lower susceptibility to the tested extracts. Among the tested pathogens, *Escherichia coli* showed the highest sensitivity, with the maximum inhibition observed for aqueous extracts of *Achyranthes aspera* (17.0 ± 1.4 mm) and *Withania somnifera* (16.0 ± 1.3 mm). The remaining extracts exhibited moderate to weak activity against *Klebsiella pneumoniae* and *Salmonella Typhimurium*, while minimal inhibitory effects were observed against *Proteus mirabilis*. Among the different extraction solvents, aqueous extracts demonstrated the strongest antibacterial performance, followed by methanol extracts, whereas ethanol extracts showed comparatively lower activity.

**Table 4B:** Medicinal plant extracts' antibacterial activity against Gram‐negative bacterial isolates (Mean inhibition zone ± SD, mm).

Plant Extract	Solvent	*Proteus mirabilis*	*Salmonella Typhimurium*	*Klebsiella pneumoniae*	*Escherichia coli*
*W. somnifera*	Methanol (80%)	1.6 ± 0.92	4.3 ± 1.18	3.3 ± 1.04	13.3 ± 1.49
	Ethanol (70%)	2.0 ± 0.81	5.3 ± 0.97	3.6 ± 0.06	14.6 ± 0.89
	Aqueous	3.0 ± 1.10	6.0 ± 1.20	5.0 ± 1.00	16.0 ± 1.30
*A. aspera*	Methanol (80%)	2.0 ± 1.00	5.0 ± 1.04	4.0 ± 0.81	15.0 ± 1.50
	Ethanol (70%)	1.0 ± 0.50	4.0 ± 1.04	3.0 ± 0.92	12.0 ± 1.20
	Aqueous	3.0 ± 1.00	4.0 ± 0.90	4.0 ± 0.80	17.0 ± 1.40
*V. sinaiticum*	Methanol (80%)	1.3 ± 1.14	3.0 ± 0.90	3.0 ± 0.70	9.6 ± 1.23
	Ethanol (70%)	1.3 ± 0.65	3.0 ± 0.80	3.0 ± 0.60	8.0 ± 1.10
	Aqueous	2.0 ± 0.90	6.0 ± 1.10	4.0 ± 0.90	12.0 ± 1.20
*Aloe vera*	Methanol (80%)	1.0 ± 0.60	3.3 ± 1.04	2.0 ± 0.81	5.6 ± 1.23
	Ethanol (70%)	1.0 ± 0.50	2.0 ± 0.80	1.0 ± 0.40	4.0 ± 0.90
	Aqueous	2.0 ± 0.70	5.0 ± 1.00	3.0 ± 0.80	8.0 ± 1.10
*C. zeylanica*	Methanol (80%)	2.0 ± 1.00	3.3 ± 1.04	3.0 ± 0.70	5.0 ± 1.41
	Ethanol (70%)	1.0 ± 0.50	2.0 ± 0.70	2.0 ± 0.60	3.0 ± 0.90
	Aqueous	3.0 ± 0.90	5.0 ± 1.00	3.0 ± 0.80	8.0 ± 1.20
Erythromycin	Positive control	15.0 ± 1.3	19.0 ± 1.7	16.0 ± 1.4	22.0 ± 1.9

*Note:* Values are presented as mean inhibition zone diameter (mm) ± standard deviation (SD).

Abbreviations: Aqueous = Water extract, E = Ethanol extract, M = Methanol extract.

### 3.4 Comparative Antibacterial Performance of the Extracts

**Figure 5:**
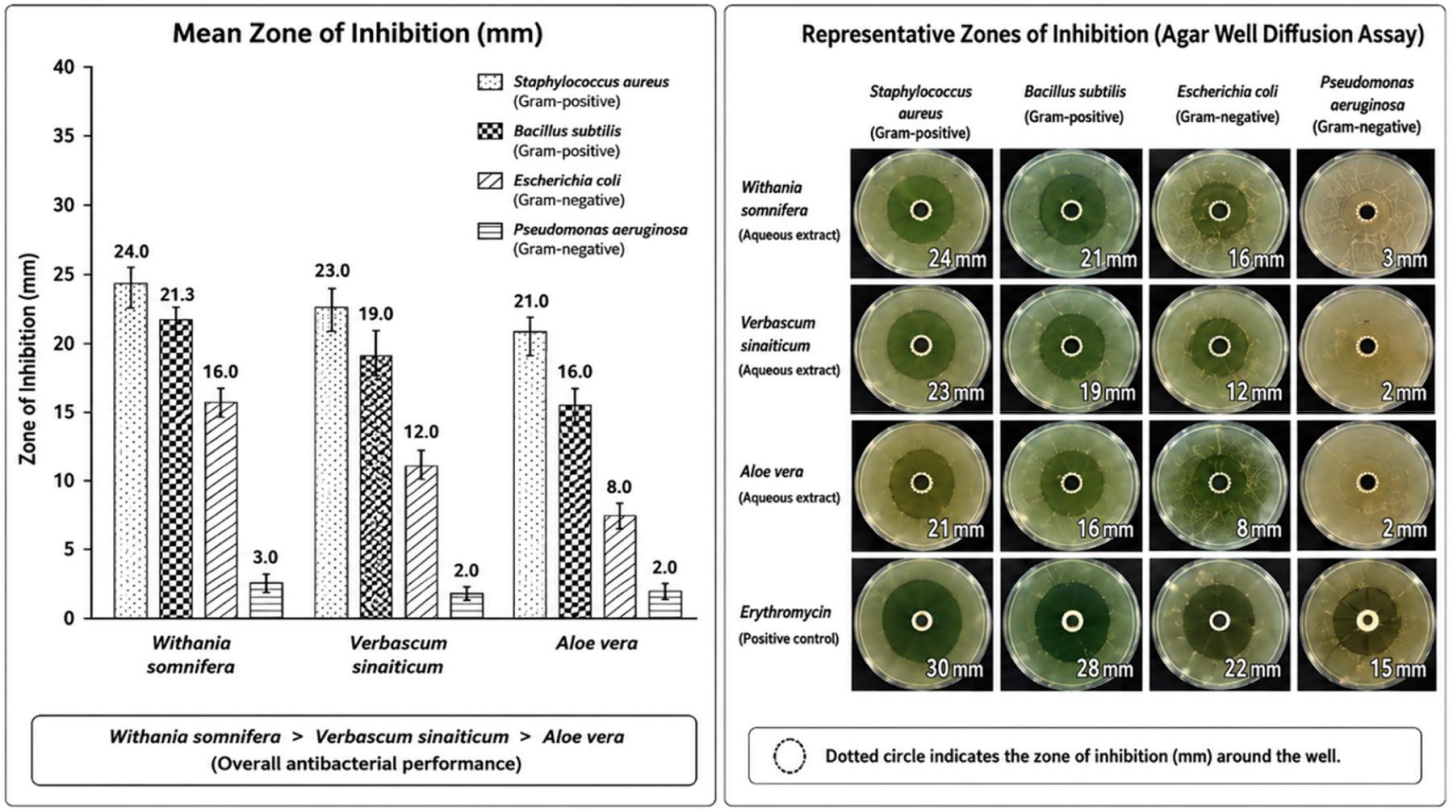
Comparative antibacterial activity and phytochemical constituents of medicinal plant extracts. The figure shows the antibacterial performance of extracts from *Withania somnifera, Verbascum sinaiticum*, and *Aloe vera* against bacterial isolates, together with representative agar well diffusion assay results and identified phytochemical constituents.

The comparative evaluation of antibacterial activity demonstrated differences among the tested medicinal plant extracts. Among the investigated plants, *Achyranthes aspera* and *Withania somnifera* exhibited comparatively stronger antibacterial effects, particularly against Gram‐positive bacterial isolates. *Verbascum sinaiticum* and *Aloe vera* also showed notable inhibitory activity, whereas *Capparis zeylanica* demonstrated relatively lower activity. The differences in antibacterial performance may be associated with variations in phytochemical composition and the efficiency of different extraction solvents in recovering bioactive compounds, as shown in (Figure 5).

## 4 Discussion

Livestock wound infections remain a major concern in veterinary medicine due to the increasing occurrence of bacterial pathogens and the growing challenge of antimicrobial resistance. In the present study, five medicinal plants traditionally used for wound healing and skin infections were evaluated for their phytochemical composition and antibacterial activity against bacterial isolates recovered from livestock wound samples. Qualitative phytochemical screening demonstrated the presence of diverse secondary metabolites, including alkaloids, flavonoids, tannins, phenolic compounds, steroids, and saponins, in the investigated plant extracts. These phytoconstituents are known to contribute to antimicrobial activity through multiple mechanisms, including disruption of bacterial cell membranes, inhibition of microbial enzymes, interference with nucleic acid synthesis, and modulation of oxidative stress pathways. Previous studies have also highlighted the contribution of phytochemical‐rich medicinal plants as potential sources of antibacterial and wound‐healing agents (Arbab 2022a; Arbab 2021a; Vitale et al. 2022).

Among the tested medicinal plants, *Withania somnifera* demonstrated strong antibacterial activity, particularly against *Staphylococcus aureus* and *Escherichia coli*. The aqueous extract of *W. somnifera* produced inhibition zones of 24 mm and 16 mm against *S. aureus* and *E. coli*, respectively. The observed activity may be associated with the presence of bioactive compounds, including *withanolides*, flavonoids, alkaloids, and phenolic constituents, which have previously been reported to possess antimicrobial, antioxidant, and anti‐inflammatory properties. Earlier studies have demonstrated that *W. somnifera* extracts exhibit inhibitory effects against a range of Gram‐positive and Gram‐negative bacterial pathogens, supporting its potential application in infection management and wound repair (Arbab et al. 2026; Arbab 2021b; Singirala et al. 2025).

The aqueous extract of *Achyranthes aspera* exhibited the highest antibacterial activity against *S. aureus*, producing an inhibition zone of 28 mm. This strong inhibitory effect may be attributed to the presence of flavonoids, tannins, alkaloids, and phenolic compounds detected during phytochemical screening. These compounds have been associated with antibacterial effects through protein precipitation, membrane destabilization, and inhibition of bacterial metabolic processes. Previous investigations have also reported the antimicrobial potential of *A. aspera* against wound‐associated bacterial pathogens, supporting its traditional use in the treatment of skin infections and wounds (Ganesh et al. 2021; Ndhlala et al. 2015).

The extracts of *Verbascum sinaiticum* and *Aloe vera* also demonstrated antibacterial activity, particularly against Gram‐positive bacterial isolates. The aqueous extracts of *V. sinaiticum* and *A. vera* showed inhibition zones of 23 mm and 21 mm, respectively, against *S. aureus*. The antibacterial activity of *A. vera* may be related to its diverse phytochemical composition, including anthraquinones, flavonoids, and phenolic compounds, which have been associated with antimicrobial and wound‐healing effects. These findings are consistent with previous reports describing the therapeutic potential of *A. vera* in skin repair and infection control (Iosageanu et al. 2024). In contrast, *Capparis zeylanica* exhibited comparatively lower antibacterial activity, which may be related to differences in phytochemical composition and the concentration of active antimicrobial constituents (Arbab 2021b; Sperandeo et al. 2009).

In this study, Gram‐positive bacteria generally showed greater susceptibility to plant extracts compared with Gram‐negative bacteria. *S. aureus* was the most susceptible pathogen, whereas *Proteus mirabilis* and *Enterococcus faecalis* showed comparatively lower sensitivity. The reduced susceptibility of Gram‐negative bacteria may be explained by structural differences in their cell envelopes. The outer membrane of Gram‐negative bacteria, which contains lipopolysaccharides, acts as an additional permeability barrier that can restrict the penetration of antimicrobial compounds. Similar observations have been reported in previous studies investigating plant‐derived antibacterial agents (Rayan et al. 2020).

The bacterial isolates obtained from the livestock wound infections revealed that *Staphylococcus aureus* was the most common bacterium, followed by *Escherichia coli* and *Staphylococcus epidermidis*. The same has been observed in a previous veterinary study, in which *S. aureus* was one of the major pathogens causing wound and skin infection in animals due to its tendency to invade damaged tissues and to evade host defense mechanisms (Foster 2009).

The extraction solvent influenced the antibacterial performance of plant extracts. In the present study, aqueous extracts generally exhibited stronger antibacterial effects than ethanol extracts, while methanol extracts showed intermediate activity. Differences in extraction efficiency may influence the recovery of bioactive compounds, particularly polar phytochemicals such as phenolics and flavonoids. Water‐based extraction may facilitate the recovery of certain antimicrobial compounds responsible for bacterial growth inhibition, which could explain the higher activity observed for several aqueous extracts. Similar solvent‐dependent variations in antibacterial activity have been reported previously (Jia et al. 2025; Negahban et al. 2026).

The bacterial distribution obtained from livestock wound samples revealed that *S. aureus* was the predominant isolate, followed by *E. coli* and *S. epidermidis*. The predominance of *S. aureus* in wound infections is consistent with previous veterinary studies, as this pathogen possesses several virulence mechanisms that facilitate colonization, tissue invasion, and persistence within damaged tissues (Arbab et al. 2022b; Di Bella et al. 2025). The presence of both Gram‐positive and Gram‐negative bacterial pathogens highlights the complexity of livestock wound infections and the need for alternative antimicrobial strategies.

Overall, the findings of this study support the traditional use of the selected medicinal plants for wound‐related infections and demonstrate their potential as sources of natural antibacterial compounds. However, further investigations are required to identify and quantify the specific bioactive molecules responsible for antibacterial activity using advanced analytical approaches such as LC–MS/MS, GC–MS, and HPLC. Future studies should also evaluate minimum inhibitory concentration (MIC), minimum bactericidal concentration (MBC), cytotoxicity, synergistic effects with conventional antibiotics, and in vivo wound‐healing efficacy to determine their clinical applicability in veterinary medicine.

## 5 Future Directions

Although the present study demonstrates the antibacterial potential of selected medicinal plant extracts against livestock wound‐associated bacterial pathogens, further investigations are required to validate their therapeutic applications. Future studies should include quantitative antimicrobial evaluations, such as minimum inhibitory concentration (MIC) and minimum bactericidal concentration (MBC) assays, to determine the precise antibacterial potency of the extracts. Bioassay‐guided fractionation and advanced phytochemical characterization using techniques such as LC–MS/MS and GC–MS should be performed to identify the specific bioactive compounds responsible for antibacterial activity.

In addition, toxicity evaluation, cytocompatibility studies, and in vivo wound‐healing models are necessary to assess the safety and therapeutic efficacy of these extracts. Further research should also investigate the mechanisms of antibacterial action, potential synergistic effects with conventional antibiotics, and the development of plant‐derived formulations for veterinary wound management.

## 6 Conclusion

The present study demonstrated that medicinal plant extracts possess antibacterial potential against bacterial pathogens isolated from livestock wound infections. Among the tested plants, *Withania somnifera* and *Achyranthes aspera* exhibited the strongest antibacterial activity, particularly against *Staphylococcus aureus*, while other plants including *Verbascum sinaiticum* and *Aloe vera* also showed notable inhibitory effects. Phytochemical screening confirmed the presence of important bioactive constituents, including alkaloids, flavonoids, tannins, phenolic compounds, steroids, and saponins, which may contribute to their antimicrobial properties. The higher susceptibility of Gram‐positive bacteria compared with Gram‐negative bacteria and the variation among extraction solvents highlight the importance of plant composition and extraction strategies in determining antibacterial efficacy. These findings support the potential application of selected medicinal plants as natural sources of antimicrobial agents for managing livestock wound infections. However, further studies involving compound identification, MIC/MBC determination, toxicity evaluation, and in vivo validation are required to establish their therapeutic potential and practical application in veterinary wound management.

## Author Contributions

**Safia Arbab:** writing – original draft, writing – review and editing, conceptualization. **Hanif Ullah:** validation, writing – review and editing. **Mian Sayed Khan:** writing – review and editing. **Khalaf F. Alsharif:** writing – review and editing. **Fuad M. Alzahrani:** writing – review and editing. **Khalid J. Alzahrani:** writing – review and editing. **Zhongyi Zeng:** writing – review and editing, funding acquisition. **Li Ka:** writing – review and editing.

## Funding

The authors have nothing to report.

## Conflicts of Interest

The authors declare no conflicts of interest.

## Data Availability

The data that supports the findings of this study are available in the supporting information of this article.
